# Adult Living Donor Liver Transplantation Across ABO-Incompatibility

**DOI:** 10.1097/MD.0000000000001796

**Published:** 2015-10-23

**Authors:** Chen-Fang Lee, Chih-Hsien Cheng, Yu-Chao Wang, Ruey-Shyang Soong, Tsung-Han Wu, Hong-Shiue Chou, Ting-Jung Wu, Kun-Ming Chan, Ching-Song Lee, Wei-Chen Lee

**Affiliations:** From the Department of Liver and Transplantation Surgery (C-FL, C-HC, Y-CW, T-HW, H-SC, T-JW, K-MC, W-CL), Department of Hepatology, Chang-Gung Memorial Hospital, Linkou, Taiwan (C-SL), Department of General Surgery, Chang-Gung Memorial Hospital, Keelung, Taiwan (R-SS); and Chang-Gung University College of Medicine, Taoyuan, Taiwan (T-JW, K-MC, W-CL).

## Abstract

The objective of this study was to evaluate the results of adult ABO-incompatible living donor liver transplantation (LDLT).

ABO-incompatible LDLT is an aggressive treatment that crosses the blood-typing barrier for saving lives from liver diseases. Although graft and patient survival have been improved recently by various treatments, the results of adult ABO-incompatible LDLT require further evaluation.

Two regimens were designed based on isoagglutinin IgG and IgM titers and the time course of immunological reactions at this institute. When isoagglutinin IgG and IgM titers were ≤64, liver transplantation was directly performed and rituximab (375 mg/m^2^) was administrated on postoperative day 1 (regimen I). When isoagglutinin titers were >64, rituximab (375 mg/m^2^) was administered preoperatively with or without plasmapheresis and boosted on postoperative day 1 (regimen II). Immunosuppression was achieved by administration of mycophenolate mofetil, tacrolimus, and steroids.

Forty-six adult ABO-incompatible and 340 ABO-compatible LDLTs were performed from 2006 to 2013. The Model for End-Stage Liver Disease scores for ABO-incompatible recipients ranged from 7 to 40, with a median of 14. The graft-to-recipient weight ratio ranged from 0.61% to 1.61% with a median of 0.91%. The 1-, 3-, and 5-year survival rates were 81.7%, 75.7%, and 71.0%, respectively, for ABO-incompatible LDLT recipients, compared to 81.0%, 75.2%, and 71.5% for ABO-C recipients (*P* = 0.912). The biliary complication rate was higher in ABO-incompatible LDLT recipients than in the ABO-compatible recipients (50.0% vs 29.7%, *P* = 0.009).

In the rituximab era, the blood type barrier can be crossed to achieve adult ABO-incompatible LDLT with survival rates comparable to those of ABO-compatible LDLT, but with more biliary complications.

## INTRODUCTION

Liver transplantation is the only effective treatment for the patients with acute or chronic liver failure. However, donor livers are always short supply and do not meet the demand for liver transplantations.^1^ Securing living donors is 1 way to expand the donor pool, particularly in Asia.^2^ Although the number of living-donor liver transplantations (LDLTs) is increasing, the shortage of organs remains. When ABO-compatible (ABO-C) donors are not available, crossing the ABO blood type barrier becomes the only chance for some patients to receive a new liver. In such circumstances, living liver donations from ABO-incompatible (ABO-I) donors become the only choice for patients who have rapid deterioration of liver function or hepatocellular carcinoma and who remain on the waiting list.^3,4^

ABO-I liver transplantations are sometimes performed under urgent conditions. The long-term results are not as good when compared with ABO-C liver transplantation.^5^ Consequently, ABO-I LDLT is considered a high-risk procedure because of the possibilities of antibody-mediated rejection, vascular thrombosis, and biliary complications.^6–8^ Since the 1990s, ABO-I LDLT has been performed at Kyoto University in Japan. They reported a 5-year survival rate for adults of only 22%, which was far inferior to that of ABO-C liver transplantation.^8^ In a recent report from the National Registry of Japan, the results of ABO-I LDLT for adults were not as satisfactory as the 5-year survival rate in infants.^7^ In 2003, the anti-CD20 monoclonal antibody (rituximab) was introduced for use in liver transplantation, and the survival rates of ABO-I liver transplantation improved greatly.^7,9^ Organ transplantation across the ABO blood type barrier became feasible.

Although ABO-I liver transplantation is now feasible, antibody-mediated rejection remains a critical problem that deteriorates the graft liver function. Researchers in Japan developed preoperative preparation with rituximab and plasmapheresis/plasma exchange to reduce anti-A/anti-B isoagglutinin titers to prevent antibody-mediated rejection and postoperative local infusion to prevent vascular occlusion.^7,10^ In western countries, a different regimen using absorb-columns was used to reduce the isoagglutinin levels.^11,12^ These preparation procedures are either complicated or expensive or both. We established a modified preparation regimen for ABO-I LDLT in 2006, which is simple and easy to perform for adult ABO-I LDLT. We examined the results of adult ABO-I LDLT performed after applying the modified preparation regimen.

## MATERIALS AND METHODS

### Patients and Grafts

From 2006 to 2013, LDLTs were performed on 386 adult patients in our institution. Among them, 46 (11.9%) patients underwent ABO-I LDLTs. The grafts, procured from relatives, were all right lobes of the liver. The middle hepatic veins were not included in the grafts. The venous outflow of segments 5 and 8 were reconstructed by cryopreserved the iliac veins when congestion was demonstrated in the right paramedian sector during perfusion with a cold histidine-tryptophan-ketoglutarate solution on the back table.^13^ All patients had duct-to-duct biliary reconstruction. Splenectomy was not performed for all patients. The hospital ethics committee approved this study (CGMH IRB No. 101-2410B).

### Pre-Transplant Preparation, Post-Transplant Immunosuppression

**FIGURE 1:**
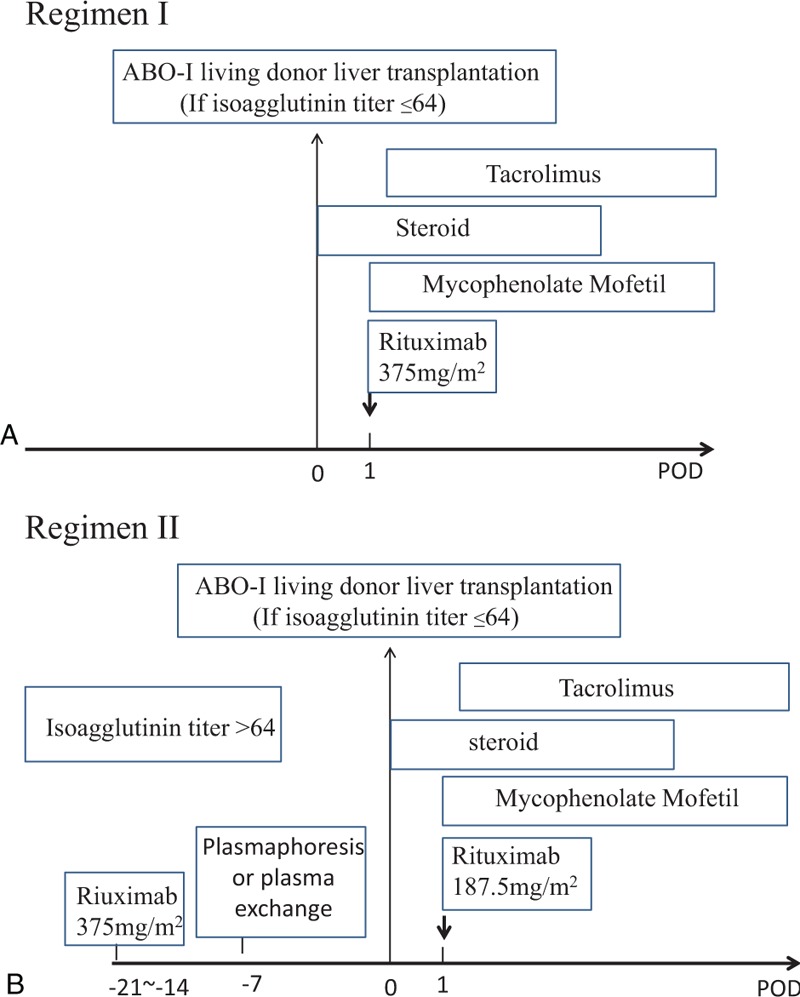
Immunomodulation protocols with rituximab, plasmapheresis or plasma exchange, and immunosuppressive regimens for ABO-incompatible (ABO-I) live donor liver transplantation (LDLT). (A) Regimen I: if IgG and IgM anti-ABO isoagglutinin titers were ≤64, patients underwent ABO-I LDLT directly. Patients received rituximab (375 mg/m^2^) on postoperative day 1. (B) Regimen II: if IgG and IgM anti-ABO isoagglutinin titers were >1:64, patients received rituximab intravenously 3 week (375 mg/m^2^) before liver transplantation. Patients then underwent plasmapheresis or plasma exchange. Finally, LDLT was performed. Patients received a rituximab boost after transplantation on postoperative day 1. ABO-I = ABO-incompatible, LDLT = live donor liver transplantation.

The acceptable criterion for antiblood type isoagglutinin titers is 1:64 or less when performing ABO-I LDLT. Depending on the anti-A and -B isoagglutinin titers before transplantation, we designed 2 different pretransplantation preparation regimens. If the IgG and IgM anti-ABO isoagglutinin titers were ≤64, then the patient underwent ABO-I LDLT directly. On postoperative day (POD) 1, rituximab (375 mg/m^2^) was administered to deplete the B-cells as prophylaxis against antibody-mediated rejection (regimen I, Figure 1A). If IgG and IgM anti-ABO isoagglutinin titers were >64, rituximab (375 mg/m^2^) was given intravenously 3 weeks before liver transplantation. The IgG and IgM titers were checked again 2 weeks after the rituximab infusion. If the isoagglutinin titers were still >64, plasmapheresis or plasma exchange were performed and repeated as needed in an attempt to decrease the anti-A or anti-B antibody titers to ≤64 at the time of LDLT. Then, LDLT was performed. On POD 1, rituximab (187.5 mg/m^2^) was administered to deplete the B-cells further (regimen II, Figure 1B). Postoperative immunosuppression was achieved with mycophenolate mofetil, tacrolimus, and steroids. Mycophenolate mofetil (1 g/day) was given orally from POD 1. Tacrolimus (2 mg/day) was started orally on POD 2 or 3 as renal function returned. The dosage of tacrolimus was adjusted according to its trough levels. Methylprednisolone, 500 mg, was given intravenously after reperfusion. Postoperatively, methylprednisolone was tapered from 200 mg/day to 40 mg/day over 5 days. The steroid was then switched to oral prednisolone 20 mg/day and stopped within 3 months after transplant.

### Diagnosis of Acute Rejection

Rejection was diagnosed by its clinical manifestations of biochemical abnormalities and marked increases in hepatic enzymes. Acute cellular rejection was diagnosed when aspartate aminotransferase (AST) and alanine aminotransferase (ALT) were elevated to twice their normal range (AST, 0–34 IU/L; ALT, 0–36 IU/L) or a rise in >30 IU/L over the previous day.^14^ Acute humoral rejection was diagnosed when serum levels of AST and ALT surged along with markedly decreased blood flow in the portal vein.^15^ Liver biopsies were reserved for the patients with persistent, abnormal liver function after initial treatments for clinically suspicious acute rejection, biliary complications, or infectious diseases.

### Biostatistics

The paired Student *t* test was used to analyze continuous variables. Categorical variables were analyzed by either the Chi-square test or Fisher's exact test. All multiple pairwise comparisons were done using the Holm–Sidak method. The survival rates were calculated using the Kaplan–Meier method and compared between groups using the log-rank test. All statistical analyses were performed with SigmaPlot 12.3 software for Windows (Systat Software, Inc, San Jose, CA). *P* < 0.05 was considered statistically significant.

## RESULTS

### Patients

Forty-six adult patients, 36 men and 10 women, underwent ABO-I LDLTs from 2006 through 2013. Their median (interquartile) age was 53.5 (49.8–60.0) years, with a range from 19 to 67 years. Their Model for End-Stage Liver Disease scores ranged from 7 to 40, with a median of 14. Their liver diseases included hepatitis B-related liver failure (n = 29, 63.0%) followed by hepatitis C-related liver failure (n = 9, 19.6%). Twenty-eight of the 46 patients (60.9%) had comorbid hepatocellular carcinoma. The median (interquartile) graft-to-recipient weight ratio was 0.91% (0.83%–1.14%), with a range from 0.61% to 1.61%. The clinical characteristics of these recipients (ABO-I LDLT) did not differ from those who underwent ABO-C LDLT (Table 1). About the blood type matching between donors and recipients, the most frequent donor to recipient mismatch of blood type was A to O (n = 17, 37.0%) followed by B to O (n = 10, 21.7%). There was no AB to O matching in this study (Table 2).

**TABLE 1:**
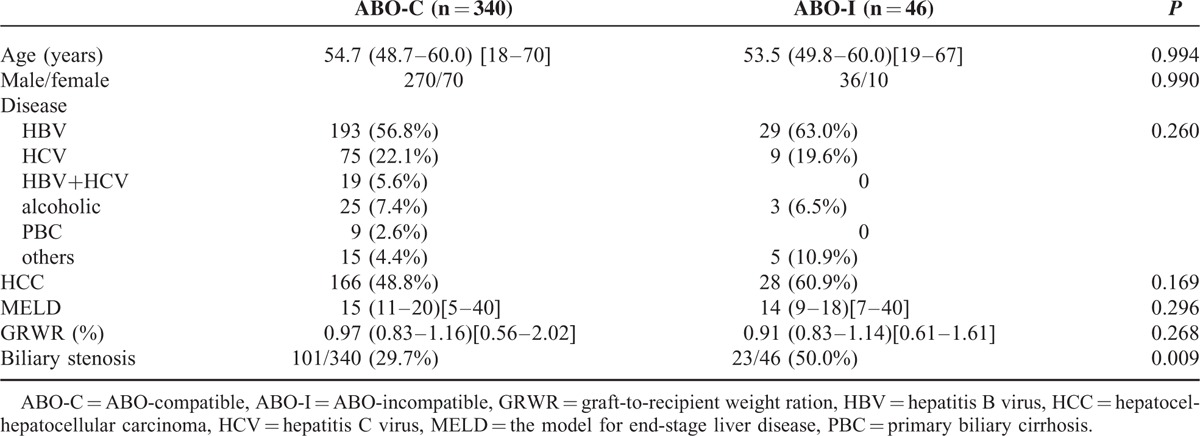
Clinical Characteristics of ABO-Compatible and Incompatible Patients

**TABLE 2:**
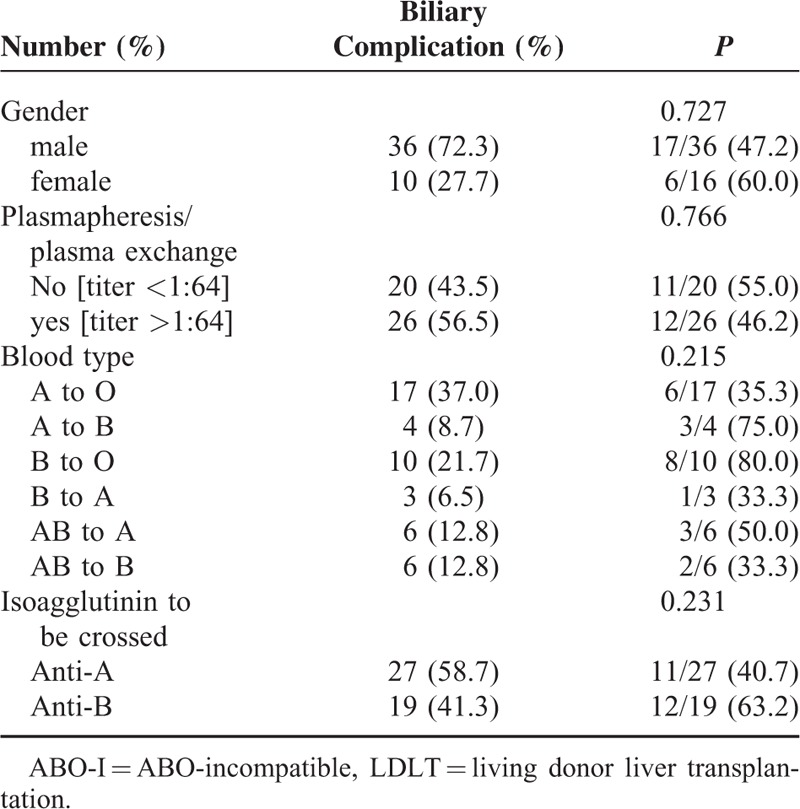
The Relationship Between Biliary Complication and Anti-A/B Isoagglutinin in ABO-I LDLT Patients

### Anti-A and -B Isoagglutinins

**FIGURE 2:**
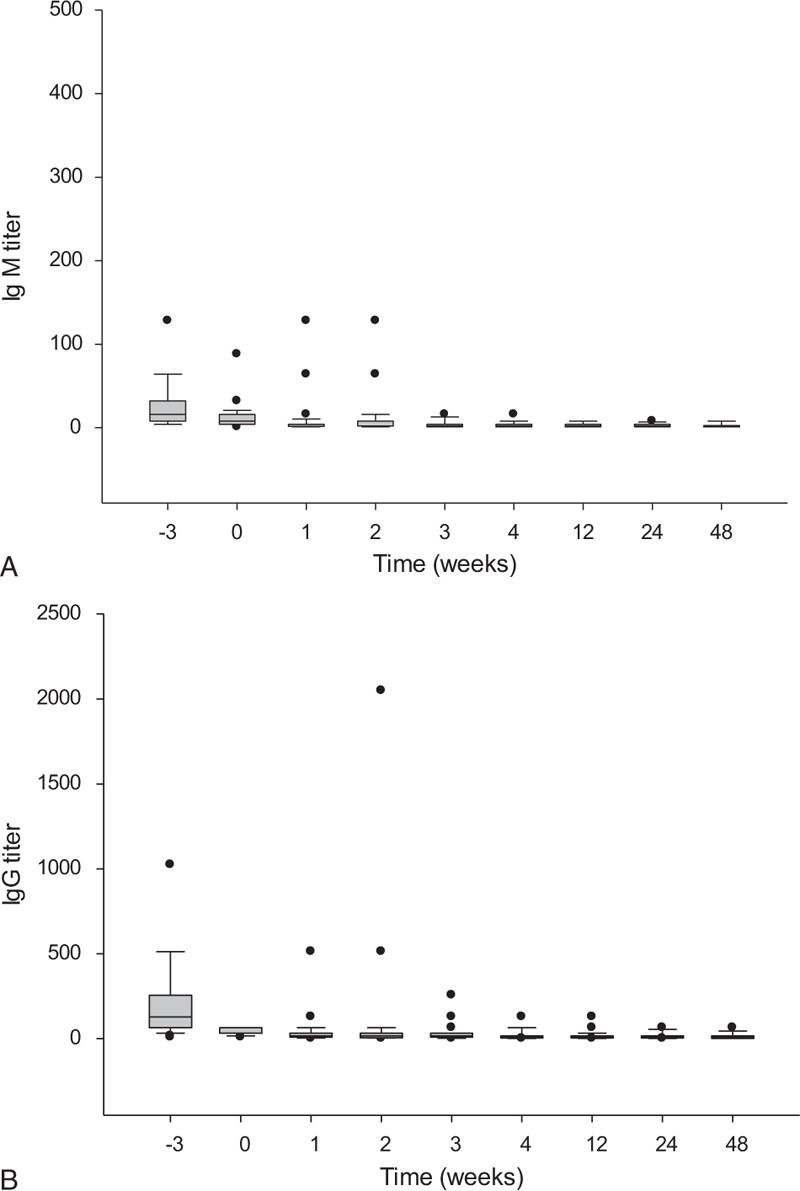
IgM and IgG anti-ABO isoagglutinin titers before and after transplantation. (A) The median (interquartile) titer of the IgM isoagglutinin was 16 (8–32), with a range from 4 to 128 before transplantation. Two weeks after transplantation, the IgM isoagglutinin titers were all ≤16. (B) The median (interquartile) titer of the IgG isoagglutinin was 128 (64–256), with a range from 8 to 1024 before transplantation. After transplantation, 3 patients experienced elevated titers >64.

**FIGURE 3:**
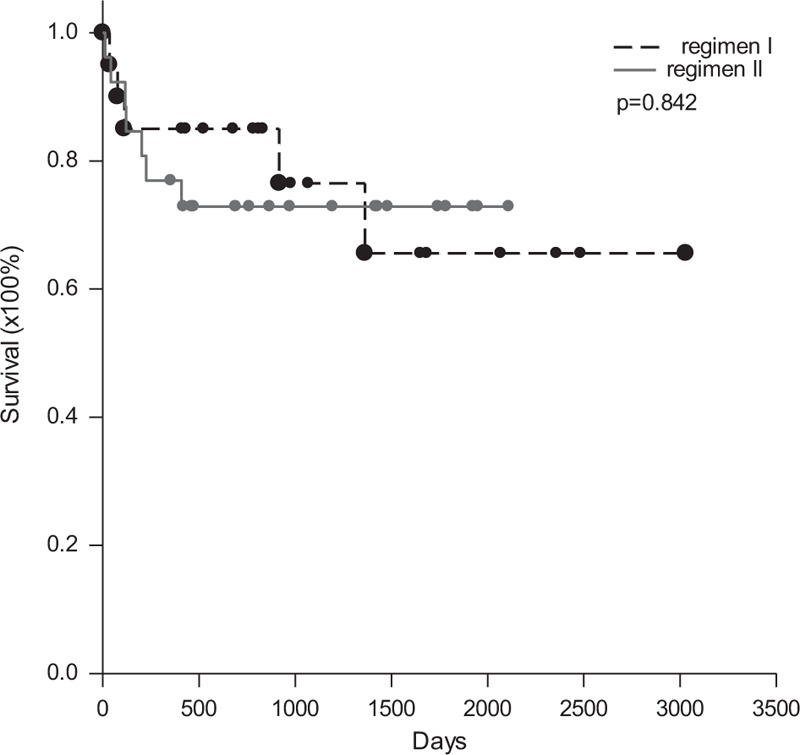
Survival rates by regimen. The 1-, 3-, and 5-year survival rates for patients treated using regimen I or II did not differ.

Before transplantation, the anti-A and -B IgM and IgG isoagglutinin titers were measured in all 46 patients undergoing ABO-I LDLT. The median (interquartile) titer of the IgM isoagglutinin was 16 (8–32), with a range from 4 to 128. The median (interquartile) titer of the IgG isoagglutinin was 128 (64–256) with a range from 8 to 1024. Among the 46 patients, the initial anti-A/B IgM and IgG isoagglutinin titers were both ≤64 in 20 (43.5%) patients. All these patients were managed according to regimen I. LDLT was performed directly, and rituximab 375 mg/m^2^ was administered on POD 1. Another 26 (56.5%) patients with anti-A and -B IgG isoagglutinin titers >64 were managed according to regimen II. Anti-A and -B IgG isoagglutinin titers decreased to ≤64 in 12 of 26 patients, and plasmapheresis and plasma exchange were not performed before transplantation. For another 14 patients with isoagglutinin titers >64, plasmapheresis or plasma exchange was performed to decrease the anti-A and -B IgG isoagglutinin titers before transplantation. The average number of courses of plasmapheresis or plasma exchange was 3.8 ± 2.0, with a range from 2 to 9. After transplantation, anti-A and -B IgM and IgG isoagglutinin titers were regularly measured. Only 2 patients (4.3%) had higher IgM isoagglutinin titers after transplantation than at transplantation. Two weeks after transplantation, the IgM isoagglutinin titers were all ≤16 (Figure 2A). For IgG isoagglutinins, 3 patients experienced elevated titers >64 within 1 month of transplantation (Figure 2B). Regardless of the management regimen used, long-term survival did not differ between the patients. The 1-, 3-, and 5-year survival rates for the patients treated with regimen I were 85.0%, 76.5%, and 65.5%, respectively, compared with 76.9%, 72.9%, and 72.9% for the patients treated with regimen II (*P* = 0.842, Figure 3).

### Tacrolimus Trough Levels

**FIGURE 4:**
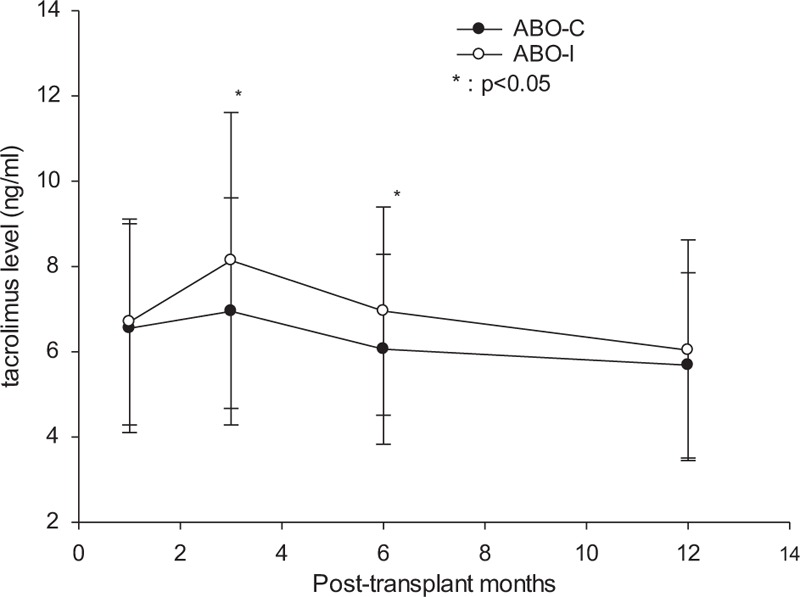
Trough levels of tacrolimus in patients that underwent ABO-I or ABO-C live donor liver transplantation (LDLT). In the first month, the trough levels of tacrolimus did not differ between ABO-I and ABO-C LDLTs. In the 3rd and 6thmonths, tacrolimus levels of ABO-I recipients were higher than those for ABO-C recipients were. At the end of the 1st year after LDLT, the trough levels of tacrolimus did not differ between the ABO-I and ABO-C recipients. ABO-C = ABO-compatible, ABO-I = ABO-incompatible, LDLT = live donor liver transplantation.

Trough levels of tacrolimus were measured and compared to the levels in the recipients with ABO-C LDLTs to determine whether ABO-I LDLT recipients needed higher levels of immunosuppressive agents to prevent rejection than the ABO-C recipients. In the first month, the trough levels of tacrolimus did not differ between ABO-I and ABO-C LDLT recipients (6.55 ± 2.45 ng/mL vs 6.70 ± 2.42 ng/mL, *P* = 0.387). In the 3rd and 6th months after transplant, the tacrolimus levels for ABO-I recipients were higher than those for ABO-C recipients were (8.14 ± 3.47 ng/mL vs 6.95 ± 2.67 ng/mL, *P* = 0.040 and 6.95 ± 2.44 ng/mL vs 6.06 ± 2.23 ng/mL, respectively; *P* = 0.015). At the end of the 1st year after transplant, the trough levels of tacrolimus between ABO-I and ABO-C LDLT recipients did not differ (5.68 ± 2.18 ng/mL versus 6.04 ± 2.59 ng/mL, *P* = 0.581) (Figure 4).

### Graft and Patient Survival

**FIGURE 5:**
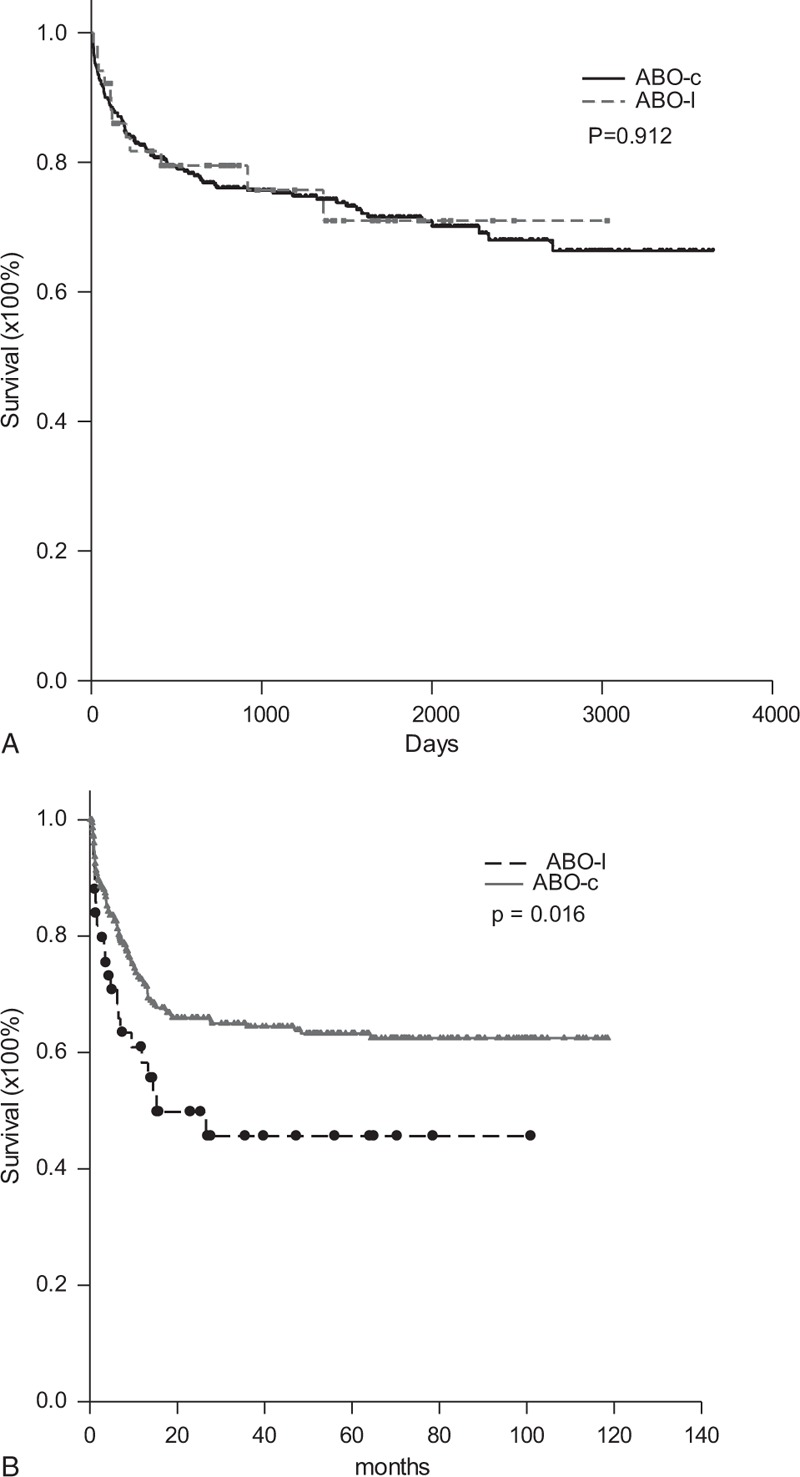
Patient and graft survival and biliary complication-free survival for ABO-I and ABO-C LDLT patients. (A) The patient and graft survival did not differ between ABO-I and ABO-C LDLT patients. (B) ABO-I LDLT had a higher biliary complication rate than did ABO-C LDLT. The 1-year and 2-year biliary complication-free survival for ABO-I LDLT recipients were 58.3% and 49.8%, respectively, compared with 72.5% and 66.0% for ABO-C LDLT recipients. ABO-C = ABO-compatible, ABO-I = ABO-incompatible, LDLT = live donor liver transplantation.

No ABO-I or ABO-C LDLT recipient underwent re-transplantation. Therefore, graft survival and patient survival were the same in this study. The long-term survival rates of the patients that underwent ABO-I LDLT were calculated using the Kaplan–Meier method and compared to the patients that underwent ABO-C LDLT. The 1-, 3-, and 5-year survival rates were 81.7%, 75.7%, and 71.0%, respectively, compared to 81.0%, 75.2%, and 71.5% for the ABO-C LDLT patients (*P* = 0.912, Figure 5A). The results showed that the survival rates for ABO-I and ABO-C LDLTs did not differ. Two recipients had clinically suspected acute humoral rejection. One of the 2 patients managed using regimen II had acute humoral rejection on POD 7. His AST suddenly spiked from 73 to 6084 U/L and his ALT rose from 145 to 2831U/L within 3 days. However, Doppler ultrasonography showed patent hepatic artery inflow, patent portal vein inflow, and patent hepatic vein outflow although portal vein flow was decreased markedly. At the same time, his IgM and IgG isoagglutinin titers increased to 64 and 2048, respectively. Despite aggressive treatment with plasmapheresis, the patient died on POD 14. The remaining patient was managed using regimen I and developed acute humoral rejection on POD 16. The clinical pictures were similar to those of the first patient. The AST and ALT suddenly spiked from 48 and 81 U/L to 2104 and 885 U/L, respectively, within 36 h. Doppler ultrasonography also showed patent hepatic artery inflow, patent hepatic outflow, and decreased portal vein inflow. Despite aggressive treatment with bortezomib and plasmapheresis, the patient's condition deteriorated and he died on POD 36. Both of the deceased patients had been re-listed for liver transplantations, but grafts were not available, and re-transplantation was not performed.

### Biliary Complications

Twenty-three (50%) recipients of ABO-I LDLTs had biliary complications, which was a much higher rate than that of ABO-C LDLT recipients (29.7%, *P* = 0.009, Table 1). All the biliary complications consisted of stenosis or bile leakage followed by stenosis at anastomotic sites. No ischemic biliary stricture with cholangitis was observed. Twenty of 23 biliary complications were successfully treated with endoscopic retrograde biliary stenting whereas the remaining 3 were treated with percutaneous trans-hepatic cholangiographic drainage. The biliary complication rate between male and female recipients did not differ (*P* = 0.727). Furthermore, the biliary complication rate between different donor-recipient ABO blood type matches did not differ (*P* = 0.215, power = 0.496, Table 2). Nonetheless, the patients with donor to recipient blood type matches of B to O had up to an 80% biliary complication rate. Considering all the patients, more patients with blood type B mismatches had biliary complications compared to patients with type A mismatches (63.2% vs 40.7%, *P* = 0.231 power = 0.208). The 1- and 2-year biliary complication-free survival rates for ABO-I LDLT recipients were 58.3% and 49.8%, respectively, compared with 72.5% and 66.0% for ABO-C LDLT recipients (*P* = 0.016, Figure 5B).

## DISCUSSION

Organ shortages remain a major problem for patients waiting for liver transplants. Because the number of deceased donors is small in Taiwan and other Eastern countries, there is often no choice but to perform LDLT. When ABO-C liver grafts are not available, utilization of grafts from ABO-I donors becomes the only available option for some patients in the critical condition.^3,4^ Recent studies have demonstrated that the results of ABO-I liver transplantation have improved because of various new therapeutic strategies.^9,16,17^ However, there are still several problems associated with ABO-I liver transplantation such as high isoagglutinin titers, application of localized hepatic rituximab infusions, long-term survival, and others.

In this study, we designed 2 easy regimens to prepare adult patients for ABO-I LDLTs. These easy-to-perform regimens involve B-cell depletion by preoperative or postoperative rituximab standard infusion rather than local infusion via the hepatic artery and portal vein. If the pre-existing isoagglutinin titers were high (>64), the B-cells were depleted by rituximab and the isoagglutinins were washed out by plasmapheresis or plasma exchange preoperatively. If the pre-existing isoagglutinin titers were ≤64, liver transplantation proceeded directly, and the B-cells were depleted by rituximab postoperatively. The isoagglutinin titers required to perform LDLT were set at ≤64 based on ABO-mismatched platelet transfusions in which the defined cut-off minimum critically high anti-A and -B Ig and IgM titers were 64.^18^ Local infusion with steroid and prostaglandin E1 via the hepatic artery or portal vein was the procedure applied previously in Japan.^19,20^ This local graft infusion method is complicated and could cause catheter-related vascular problems such as thrombosis, bleeding, or catheter dislocation.^7,21^ Song et al reported that localized graft infusion caused catheter-related complications. However, they achieved successful outcomes for adult ABO-I LDLT using their desensitized protocol without local graft infusion and splenectomy.^22^ We elected not to use the local infusion technique, and no vascular thrombosis was noted. Additionally, splenectomies not performed included in our regimen. The Kyoto group reported that early prophylaxis with rituximab could take the place of splenectomy.^23^ Splenectomy increases the risk of sepsis in patients undergoing heavy immunosuppression regimens and plasma exchange.^24,25^ Therefore, our regimens for ABO-I liver transplantation are easy and do not risk the success of graft survival. Regimen I may also be applied to urgent operations in which pre-transplant preparation is not possible.

Our regimens emphasize postoperative administration of rituximab. The satisfactory depletion of B-lymphocytes and suppression of serum isoagglutinin titers are the major steps concerns in preventing acute humoral rejection. Rituximab, a chimeric anti-CD20 monoclonal antibody, is approved as a therapeutic agent for B-cell lymphoma.^26^ Because all cells of B-cell lineage express CD20 antigen except pro-B cells and plasma cells, anti-CD20 antibody depletes activated B-cells.^27–30^ It has been used for solid organ transplantations to prevent acute humoral rejection. Thus, preoperative administration of rituximab significantly lowered the postoperative isoagglutinin titers with favorable outcomes.^7,21,27,31^ However, B-cells only become activated after they encounter allografts after transplantation. Some B-cells may escape rituximab depletion preoperatively and become activated B-cells producing antibody after transplantation. Therefore, we emphasize that rituximab should be administered or boosted postoperatively in every patient to delete activated B-cells completely.

The survival rate of ABO-I LDLT was compatible with that of ABO-C LDLT. In this study, the 1-, 3-, and 5-year survival of ABO-I LDLTs were almost the same as those of ABO-C LDLTs. In a large series of Japan patients, the results of adult ABO-I LDLTs were inferior to those of infants and teenagers.^7^ After rituximab became available for use in ABO-I LDLTs, the outcomes began improving.^9^ Our ABO-I LDLT program began in the rituximab era, and the outcomes of ABO-I and ABO-C LDLTs did not differ. Thus, the ABO blood type barrier is no longer an obstacle to achieving a successful liver transplantation.

Although the long-term adult survival between ABO-I and ABO-C LDLTs was similar, the biliary complication rate for ABO-I LDLT was higher than that of ABO-C LDLT in our study. The epithelium of the bile ducts and endothelium of the hepatic artery and portal vein express ABO antigens, which served as targets for antibody-mediated rejection.^32^ In our study, no hepatic artery thrombosis occurred. Therefore, we found no ischemic biliary complications. The high rate of biliary stenosis at anastomotic sites in ABO-I LDLTs could have been due to ABO immunological reactions at the sites.

Interestingly, the anti-B antibody caused 63.2% of the biliary complications compared to 40.7% of biliary complications for the anti-A antibody. Nonetheless, the mechanism for this result is not clear. Further studies are needed to explore the relationship between blood type and biliary complications. As time elapsed, the incidence of biliary complication decreased. The low ABO antibody titers and the reduction of immunosuppressant therapy several months after transplantation indicated graft accommodation and successful ABO barrier management.

Acute humoral rejection occurred in 2 patients. One of the patients experienced a prominent rebound of isoagglutinin titers (1:2048) after transplantation although the IgG titer was reduced from 1024 to 64 before transplantation and stayed low in the early days after transplantation. The high-titer isoagglutinin rebound could be related to plasma cells. Recently, bortezomib, a proteasome inhibitor, has been used to treat antibody-mediated rejection and acute cellular rejection.^33^ Bortezomib could reduce or eliminate donor-specific antihuman leukocyte antigen antibody by depletion of plasma cells.^34,35^ A proteasome inhibitor may be included in the protocol for the patients with high IgG isoagglutinin levels when preparing for ABO-I LDLTs. A well-designed clinical trial is needed to define the role of proteasome inhibitors in liver transplant recipients.

In conclusion, the long-term results of adult ABO-I LDLTs were almost the same as for ABO-C LDLTs when rituximab is administered after (or before and after) transplantation. The survival results are encouraging. The ABO blood type barrier can be crossed to achieve successful LDLTs. A high biliary complication rate and the occurrence of acute humoral rejection imply that further studies are needed to understand post-transplantation immunological reactions in ABO-I LDLTs.
